# Treatment of negative symptoms in schizophrenia: a challenge for clinical research

**DOI:** 10.1007/s00406-023-01595-w

**Published:** 2023-03-29

**Authors:** Andrea Schmitt, Isabel Maurus, Peter Falkai

**Affiliations:** 1 Department of Psychiatry and Psychotherapy, University Hospital, LMU Munich, Nussbaumstrasse 7, 80336 Munich, Germany; 2 Laboratory of Neuroscience (LIM27), Institute of Psychiatry, University of Sao Paulo, São Paulo, Brazil; 3 Max Planck Institute of Psychiatry, Munich, Germany

Schizophrenia is a severe neuropsychiatric disease that typically emerges in late adolescence and young adulthood, persisting throughout life, and striking about 1% of the population. The high disease burden [1] is based on frequent hospitalizations, high levels of disease-related incapacity to work and early retirement. Furthermore, a substantial proportion of patients (30–50%) experience an unfavorable disease course and so-called residual symptoms, e.g., negative symptoms that remain after the acute treatment period. These symptoms respond only limited to treatment with psychotherapy or medication with antipsychotics and cause disability in everyday life, including functional impairments that prevent social and professional reintegration [2].

In this issue of European Archives of Psychiatry and Clinical Neuroscience, Wang et al. [3] applied different rating scales of negative symptoms in a large cohort of patients with schizophrenia and used network analysis to assess the correlation between negative symptom domains. The Self-evaluation of Negative Symptoms (SNS) nodes clustered together, while the Scale for Assessment of Negative Symptoms (SANS) and Brief Negative Symptom Scale (BNSS) were interrelated. Some subdomains such as anhedonia–asociality and affective flattening, both represented in the SANS, had the highest node strength. Overall, the three scales showed domain-specific correspondence and cover the complex psychopathology in schizophrenia. Since the neurobiological background of negative symptoms is widely unknown, Bayrakçi et al. [4] examined Diffusion Tensor Imaging (DTI)-based white matter architecture in schizophrenia patients compared to healthy controls and correlated network metrics with severity of negative, positive and cognitive symptoms using BNSS, the Scale for the Assessment of Positive Symptoms (SAPS) and neurocognitive tests. They found higher modularity, lower rich connections and lower nodal degree of the left thalamus and left putamen in schizophrenia patients compared to controls. In the patient group, higher modularity was correlated with negative symptoms but not with positive symptoms and cognitive deficits suggesting an alteration in modularity being specific to negative symptoms. White matter network activity may play a role in negative symptoms in schizophrenia and could be related to cell-specific pathology such as oligodendrocyte and myelination deficits in white matter tracts. It is important to disentangle neurobiological mechanisms underlying negative symptoms to develop new mechanism-based treatment strategies in schizophrenia.

In a recent meta-analysis of effects of the non-invasive brain stimulation method intermittent theta-burst stimulation as add-on therapy to antipsychotics in schizophrenia, 13 double-blind sham-controlled studies with 524 patients were included [5]. Add-on treatment is associated with improvement of negative symptoms and general psychopathology, but not with positive or cognitive symptoms. The strongest effect was detected after stimulation of the left dorsolateral prefrontal cortex, which has been related to the pathophysiology of negative symptoms. A meta-analysis of a pharmacological approach with N-Acetylcysteine as add-on therapy also revealed improvement of negative symptoms, total psychopathology and working memory. However, due to two outlier studies and the overall low number of clinical studies results have to be considered as preliminary [6].

A better evaluated and promising add-on treatment in schizophrenia is aerobic exercise. In this issue, Habelt et al. [7] explored features of behavioral addiction in alpine mountaineering, but address also that regular exercise improves physical well-being and mood and reduces anxiety. Reduced physical activity is a hallmark of schizophrenia and during the last decade the impact of physical activity and different exercise modalities on symptoms of schizophrenia have been investigated in more detail. In a meta-analysis of 27 experimental and observational studies in schizophrenia, higher levels of physical activity were associated with lower levels of negative and positive symptoms as well as general psychopathology [8]. According to a meta-analysis of 17 randomized, controlled clinical trials comprising 954 patients with schizophrenia, aerobic exercise reduced negative and positive symptoms, while non-aerobic interventions did not [9]. However, effect size was small and effects on primary negative symptoms, which are not drug induced, were not assessed separately. In an aerobic exercise study combining a 3-month endurance training with cognitive remediation, a significant effect on severity of negative symptoms and cognitive function, including verbal memory, was observed from week 6 to the end of the 3-month training period [10]. Currently, larger, multicenter studies are under way which assess effects of aerobic exercise on specific symptom and cognitive domains, and relate the psychopathology to regenerative effect of training in specific brain regions.
